# Tricuspid Valve Endocarditis in an Intravenous Drug Abuser Masquerading as Pulmonary Tuberculosis

**DOI:** 10.4103/1995-705X.76805

**Published:** 2010

**Authors:** Prashanth Panduranga, Mohammed Al-Mukhaini, Kadhim Sulaiman, Seif Al-Abri

**Affiliations:** Department of Cardiology, Royal Hospital, Muscat, Sultanate of Oman; 1 Department of Medicine, Royal Hospital, Muscat, Sultanate of Oman

**Keywords:** Intravenous Drug Abuse, Infective endocarditis, Right-sided Endocarditis, Staphylococcus aureus, Septic Pulmonary Emboli, Tricuspid valve endocarditis

## Abstract

Intravenous drug abuse contributes to considerable illness burden in developed and developing countries. Tricuspid valve endocarditis (TVE) is rare in Middle East countries, though many reports of it in intravenous drug abusers are found in other countries. We describe a case of TVE mimicking pulmonary tuberculosis in a 33-year-old man with a history of intravenous heroin use.

## INTRODUCTION

Drug abuse and addiction are major burdens to society. The economic, social, psychological and health consequences are quite devastating. Infective endocarditis (IE) is a recognized complication of intravenous drug abuse. Hundreds of articles have appeared discussing the epidemiologic, clinical and prognostic features of this entity. Although numerous case reports of tricuspid valve endocarditis (TVE) exist, reports of TVE in Middle Eastern countries are rare.

## CASE PRESENTATION

A 33-year-old male, intravenous drug abuser presented with history of fever, rigors, night sweats, cough, pleuritic chest pain, hemoptysis, and worsening dyspnea of two months duration. He had a history of pulmonary tuberculosis in 2004 and had completed treatment. He was seen by tuberculosis specialists and was again initiated on anti-tuberculosis treatment based on bilateral pulmonary infiltrates, but his sputum was negative for acid fast bacillus on three occasions.

**Figure 1:**
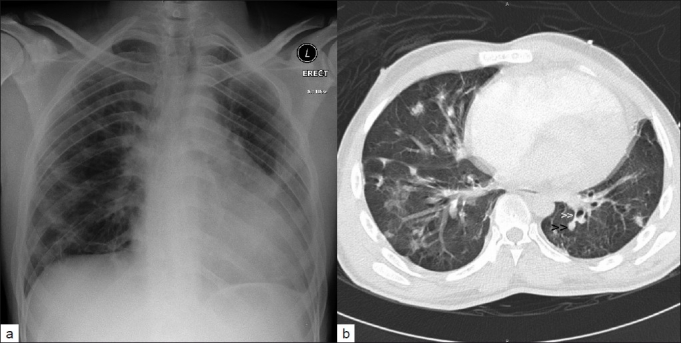
(a) Chest X-ray showing cardiomegaly with bilateral lung infiltrates and (b) CT scan of chest showing bilateral irregular pulmonary infiltrates with multiple nodules (black arrowheads) with distinct central feeding vessel (white arrowheads), consistent with septic pulmonary emboli in a patient with intravenous drug abuse and tricuspid valve endocarditis

Clinically, he was toxic, tachycardic, tachypneic, febrile with elevated juguler venous pressure, and a prominent V wave. There was a grade 3/6 pansystolic murmur over the left sternal border, bilateral scattered crepitations, hepatomegaly, and mild pedal edema. Chest X-ray showed bilateral pulmonary infiltrates. Computed tomography (CT) showed bilateral irregular pulmonary infiltrates as well as spherical nodules, and feeding vessels [Figure 1a–b]. Laboratory work-up showed leucocytosis with neutrophilia. HIV virus and hepatitis B antigen were negative and hepatitis C virus was positive.

**Figure 2:**
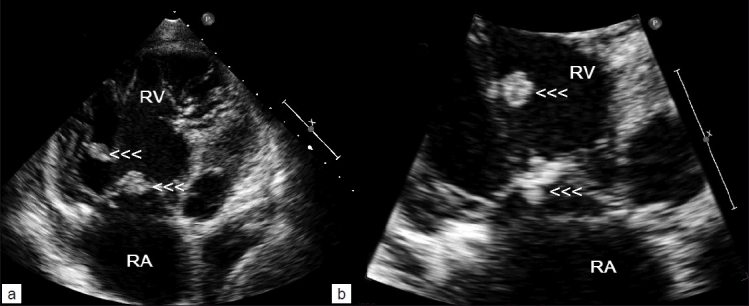
Transthoracic echocardiography (a and b) showing multiple vegetations attached to tricuspid valve leaflets (arrowheads) and one large vegetation on the chordae of anterior tricuspid leaflet (upper arrowheads) in a patient with IV drug abuse and septic pulmonary emboli. RA, right atrium; RV, right ventricle

Transthoracic echocardiography showed dilated right atrium (RA) and right ventricle (RV) with a large, oscillating vegetation attached to the anterior tricuspid valve leaflet chordate [Figure 2a–b]. There were multiple small vegetations attached to tips of both tricuspid valve leaflets. There was severe tricuspid regurgitation with calculated right ventricular systolic pressure of 40 mmHg, mild circumferential pericardial effusion, and right ventricular dysfunction. The left side of heart was normal.

Three sets of blood culture were positive for methicillin-sensitive *Staphylococcus aureus*. He was treated with intravenous flucloxacillin for 4 weeks. After a week of antibiotic therapy, he was afebrile and clinically stable, but repeated echocardiograms showed persistence of vegetation, >2 cm with severe tricuspid regurgitation, and right ventricular dysfunction. He underwent tricuspid valve replacement using size 29 Perimount bioprosthetic valve with successful recovery.

## DISCUSSION

IVDA is a social, health, and economic burden in developed and developing countries.[1] Overdose, cutaneous complications, pulmonary embolism, infective endocarditis (IE), community-acquired pneumonia, pulmonary tuberculosis, septicemia, and the transmission of blood-borne infections are well-known complications of IVDA. It has been estimated that there are 13 million IVDAs in 130 countries worldwide, of whom 10 million live in developing and transitional countries.[1] From the available data, the prevalence in eastern Europe and Asia, is 0.65% whereas in the Middle East and Africa, it is 0.2%, which is low.[1]

Right-sided endocarditis accounts for 5 to 10% of all IE and commonly involves the tricuspid valve.[2] The rarity of tricuspid valve endocarditis (TVE) are attributed to infrequent occurrence of right-sided congenital and rheumatic heart diseases as well as the low pressure, and low oxygen saturation in the right side, which protects the tricuspid and pulmonary valves from being subjected to being excessive strained.[3] TVE however, occurs predominantly in IVDAs, patients with pacemakers or defibrillators or central venous lines, those on hemodialysis and those with congenital heart diseases.[2,3] The cause for increased prevalence of TVE in IVDAs is not fully elucidated. Damage to the right-sided valves from injected particulate matter, contaminated drug solutions, and immune abnormalities are some of the causes for TVE in IVDAs.[2,4]

The exact incidence of IE in IVDAs is unknown. Acute infection is responsible for 60% of hospital admissions among IVDAs and IE is implicated in 5 to 15% of these cases.[5,6] In a Finnish study, 7.7% of the IE patients were IVDAs.[7] In the United States, the incidence is estimated at 1.5 to 3.3 cases per 1000 person-years.[5] Human immunodeficiency virus infection is common in IVDAs, with a reported prevalence of 30 to 70%.[8,9] It is also estimated that the incidence of IE in IVDAs is 2 to 5% per year and overall death rate is 5 to 10%.[9]

The presenting clinical manifestations of right- and left-sided endocarditis differ. In right-sided endocarditis, the usual manifestations are persistent fever, bacteremia, and multiple pulmonary emboli.[2,4] Hence, pleuritic chest pain, dyspnea, cough, and hemoptysis may be the presenting features, which was the case in our patient. The occurrence of peripheral emboli or neurologic symptoms in an IVDA should raise strong consideration of either left-sided endocarditis or paradoxical embolism.[4] The majority (80%) of these patients are 20 to 40 years old and male, the sex ratio being 4 to 6:1.[5] It is difficult to predict the presence of IE from the history and physical examination findings alone.[10] Cocaine use increases the occurrence of IE, possibly due to much a higher frequency of injections than in heroin users.[4,5] In the case we presented, there was a history of prolonged fever associated with other pulmonary symptoms, which were interpreted as recurrence of a previous history of tuberculosis.

Other important features of TVE include the absence of underlying heart disease in two-thirds of the patients. Only 35% of IVDAs with IE demonstrate heart murmurs on admission. Thirty percent (30%) have pleuritic chest pain and pulmonary findings may dominate the clinical picture. In 75 to 85% of the patients, chest X-ray or CT will document abnormalities such as pulmonary obstruction, infiltrates, nodules, or wedge-shaped opacities with or without cavitation, and abscesses suggesting septic emboli.[2–6,9] Almost two-thirds have extravalvular sites of infection, which are helpful in the diagnosis.[6]

The most reliable predictors of IE in febrile IVDAs are visualization of vegetations by echocardiography and the presence of embolic phenomena.[10] Tricuspid vegetations are large due to the low pressure in right heart chambers, allowing them to grow and may be in excess of 2 cm.[4] Embolized vegetations may be seen floating free in the right ventricle or pulmonary artery or maybe entrapped in the tricuspid chordal apparatus.[4] The finding of typical echocardiographic features involving right heart structures in the presence of positive blood cultures with a typical organism should be regarded as diagnostic of right-sided endocarditis, as was the case in our patient.[4] Our patient had no underlying or history heart disease; pulmonary findings dominated the clinical picture. A murmur, which may have been absent initially, was present on admission to our hospital. It has been noted that right-sided murmurs are more difficult to detect.[4] In our patient, transthoracic echocardiography identifien the tricuspid valve vegetation, confirming that transthoracic echocardiography remains an easy and highly sensitive first-line examination for the diagnosis of TVE. The interesting element in our patient was the large vegetation attached to the chordae of the anterior tricuspid valve leaflet by a pedicle, which has not been reported previously.

Our patient exhibited multiple septic emboli. Septic pulmonary emboli may cause pulmonary infarction, pulmonary abscesses, bilateral pneumothoraces, pleural effusions, mycotic aneurysms of pulmonary arteries, and empyema.[2,4] Right heart failure is rare, but can be caused by the increase of pulmonary pressures or severe right-sided valvular regurgitation or obstruction.[2] Multiple pulmonary emboli along with tricuspid valve destruction and severe tricuspid regurgitation may result in right-sided chamber volume overload/dilatation, and right heart failure.[4] Paravalvular abscess formation occurs infrequently. Hypoxemia and paradoxical embolism can occur due to right to left shunting through a patent foramen ovale.[4] Among the valves, the tricuspid valve is the most frequently affected (60–70%), followed by the mitral and aortic valves (20–30%).[9]

Although IE in IVDAs is commonly caused by *S. aureus* (60-90%), *Pseudomonas aeruginosa*, other gram-negative bacilli, polymicrobial infections, fungi, and group B streptococci have been implicated.[2–6,9] In one study, the incidence of IE was 17% among all staphylococcal bacteremia patients and 46% among IVDAs.[6] In another study, 24% of IVDAs developed methicillin-resistant *S. aureus*, with 41% of them developing IE.[11]

TVE generally has a benign prognosis, and in-hospital mortality is less than 10%.[2–6] Uncomplicated TVE is successfully treated medically in 80% of patients; however, in the remaining 20%, surgical treatment is required.[5,12] Right-sided endocarditis, no matter how severe, often allows time for medical treatment to take effect because tricuspid and pulmonary regurgitation are well-tolerated.[12] Hence, it is recommended to treat the patient medically with antibiotics initially before sending the patient to surgery.[12] In one study the main predictors of death in right-sided IE in IVDAs are vegetation size (>20 mm) and fungal etiology.[13]

The operative indications for TVE in the active stage are: 1) right heart failure secondary to severe tricuspid regurgitation with poor response to diuretic therapy; 2) IE caused by organisms which are difficult to eradicate (e.g. persistent fungi); 3) bacteremia for at least 7 days (e.g. *S. aureus, P. aeruginosa*) despite adequate antimicrobial therapy, and 4) tricuspid valve vegetations >20 mm which persist after recurrent pulmonary emboli with or without concomitant right heart failure.[2] Surgical strategies for TVE include vegetectomy with valve repair/reconstruction or replacement (either mechanical or bioprosthetic valves), even though patients addicted to IVDA are at higher risk to become reinfected, and compliance to long-term anticoagulation is unpredictable.[12] In a few studies, both mechanical and bioprosthetic valves have similar 15-year survival (47.8% for mechanical vs 46.7% for bioprosthetic valves) and re-operation-free survival (53% for mechanical vs 52% for bioprosthetic valves).[14,15]

## CONCLUSION

The clinical manifestations of right-sided endocarditis could mimic pulmonary inflammatory disease, especially if the patient has a past history of chronic lung infection such as TB as was the case in our patient. When an IVDA patient presents with fever, right-sided endocarditis is a primary diagnosis and should be ruled out early. Echocardiography is a fast, quick, and economical, and accurate diagnostic tool in such setting.”
